# The requirement of SEPT2 and SEPT7 for migration and invasion in human breast cancer via MEK/ERK activation

**DOI:** 10.18632/oncotarget.11402

**Published:** 2016-08-19

**Authors:** Nianzhu Zhang, Lu Liu, Ning Fan, Qian Zhang, Weijie Wang, Mingnan Zheng, Lingfei Ma, Yan Li, Lei Shi

**Affiliations:** ^1^ Institute of Cancer Stem Cell, Cancer Center, Dalian Medical University, Dalian, 116044, Liaoning, P.R.China; ^2^ College of Basic Medical Sciences, Dalian Medical University, Dalian, 116044 Liaoning, P.R.China; ^3^ Department of Gynecology and Obstetrics, Dalian Municipal Central Hospital Affiliated to Dalian Medical University, Dalian, 116033, Liaoning, P.R.China; ^4^ The First Affiliated Hospital of Dalian Medical University, Dalian, 116011, Liaoning, P.R.China; ^5^ State Key Laboratory of Drug Research, Shanghai Institute of Materia Medica, Chinese Academy of Sciences, Shanghai, 201203, P.R.China

**Keywords:** septin, forchlorfenuron, breast cancer, invasion, MAPK

## Abstract

Septins are a novel class of GTP-binding cytoskeletal proteins evolutionarily conserved from yeast to mammals and have now been found to play a contributing role in a broad range of tumor types. However, their functional importance in breast cancer remains largely unclear. Here, we demonstrated that pharmaceutical inhibition of global septin dynamics would greatly suppress proliferation, migration and invasiveness in breast cancer cell lines. We then examined the expression and subcellular distribution of the selected septins SEPT2 and SEPT7 in breast cancer cells, revealing a rather variable localization of the two proteins with cell cycle progression. To determine the role of both septins in mediating malignant behavior of cancer cells, we used RNA silencing to specifically deplete endogenous SEPT2 or SEPT7 in highly invasive breast cancer cell line MDA-MB-231. Our findings showed that SEPT2/7 depletion had virtually identical inhibitory effects on cellular proliferation, apoptosis, migration and invasion. Moreover, the opposite performance in migration and invasion was observed after enforced expression of SEPT2/7 in the same cell line. We further demonstrated MEK/ERK activation, but not other MAPKs and AKT, was positively correlated with the protein levels of SEPT2 and SEPT7. Additionally, in SEPT2/7-overexpressing cells, the MEK specific inhibitor U0126 was able to correct the high active status of MEK/ERK while normalizing the increased invasive behaviors of these cells. Taken together, these results strongly suggest that SEPT2 and SEPT7 are involved in breast carcinogenesis and may serve as valuable therapeutic targets for breast cancer.

## INTRODUCTION

Septins constitute a family of highly conserved GTP-binding proteins from yeast to human, and by far 14 members have been identified in human genome [1]. Septins are categorized as the fourth cytoskeletal proteins and can assemble into higher-order cytoskeletal structures including filaments, bundles and rings by heteromeric complex formation [2]. Basic elements for septin core structure have been described by forming non-polar SEPT2/6/7 hexamer [3], although the components might be variable depending on cell type and level of expression. An emerging body of studies reveals that septins maintain a conserved role in cytokinesis and have also been implicated in a variety of other cellular functions such as chromosome segregation, DNA repair, cell polarization, migration and apoptosis [1, 4–7]. In addition, septins are reported to be tightly associated with human physiological and pathological events, including bacterial infection, Alzheimer disease, Parkinson disease, and male infertility [8].

Currently, numerous studies have indicated that alterations of septin proteins, by mutation or expression changes, strongly link with tumorigenesis [1, 9–11]. Among the septin family, perhaps the best-studied member closely associated with human cancers is SEPT9, which was identified as an oncogene in ovarian, head and neck and prostate cancer cells [12]. Moreover, promoter methylation of *SEPT9* was regarded as a reliable and specific biomarker for the early detection of colorectal cancer [13]. As another exemplar of septin with oncogenic activity, downregulation of SEPT2 expression would contribute to PPARγ activation and thus suppress hepatoma cell growth [14]. Additionally, some of the most convincing evidence from the study of gliomas supports a tumor suppressor role for SEPT7 through negative regulation of the crucial cell-cycle regulators such as cyclin D1 and CDK4 [15]. These findings suggested that the septins might be critical proteins in the development of certain cancers and merit deeply exploration to further disclose the mechanisms whereby they function.

Breast cancer is the most commonly diagnosed cancer and the leading cause of cancer-related death among women worldwide [16]. Despite improvement in overall survival of breast cancer patients, drug resistance remains a huge challenge for clinical therapy and crucial for disease recurrence and progression. Therefore, the existing situation necessitates an extensive search for novel bio-molecules that may be acted as safe and effective drug targets. Cytoskeleton proteins are being developed as a new class of therapeutic targets for breast cancer. For instance, inhibition of microtubule by paclitaxel and its semi-synthetic derivatives docetaxel and cabazitaxel, which have been widely applied to chemotherapy of breast cancer, had a decent effect on preventing cancer cell mitosis and cytokinesis [17]. High expression levels of four septins (SEPT2, 8, 9 and 11) were verified in paclitaxel (Taxol) resistant MDA-MB-231 cells [18], which suggests the possibility that septins can modulate microtubule-based breast cancer chemotherapy by their guidance towards microtubule and actin cytoskeleton dynamics, which have been quite well described in epithelial cells [19] and neuron cells [20].

Given the importance of septins as non-conventional cytoskeletons in the regulation of cytokinesis and mitosis in various human cancers, we hypothesized septins are likely to be involved in the pathogenesis of breast cancer by modulating malignant phenotypes of cancer cells. Moreover, the basic information concerning the role of septin family and representative members in affecting breast cancer cell biology is at present largely unknown. Herein, this study aimed at determining whether septin proteins could influence breast cancer cell biological behavior, including cell proliferation, migration and invasion, and uncovering its underlying mechanism.

## RESULTS

### FCF suppress breast cancer cell proliferation and invasion

**Figure 1:**
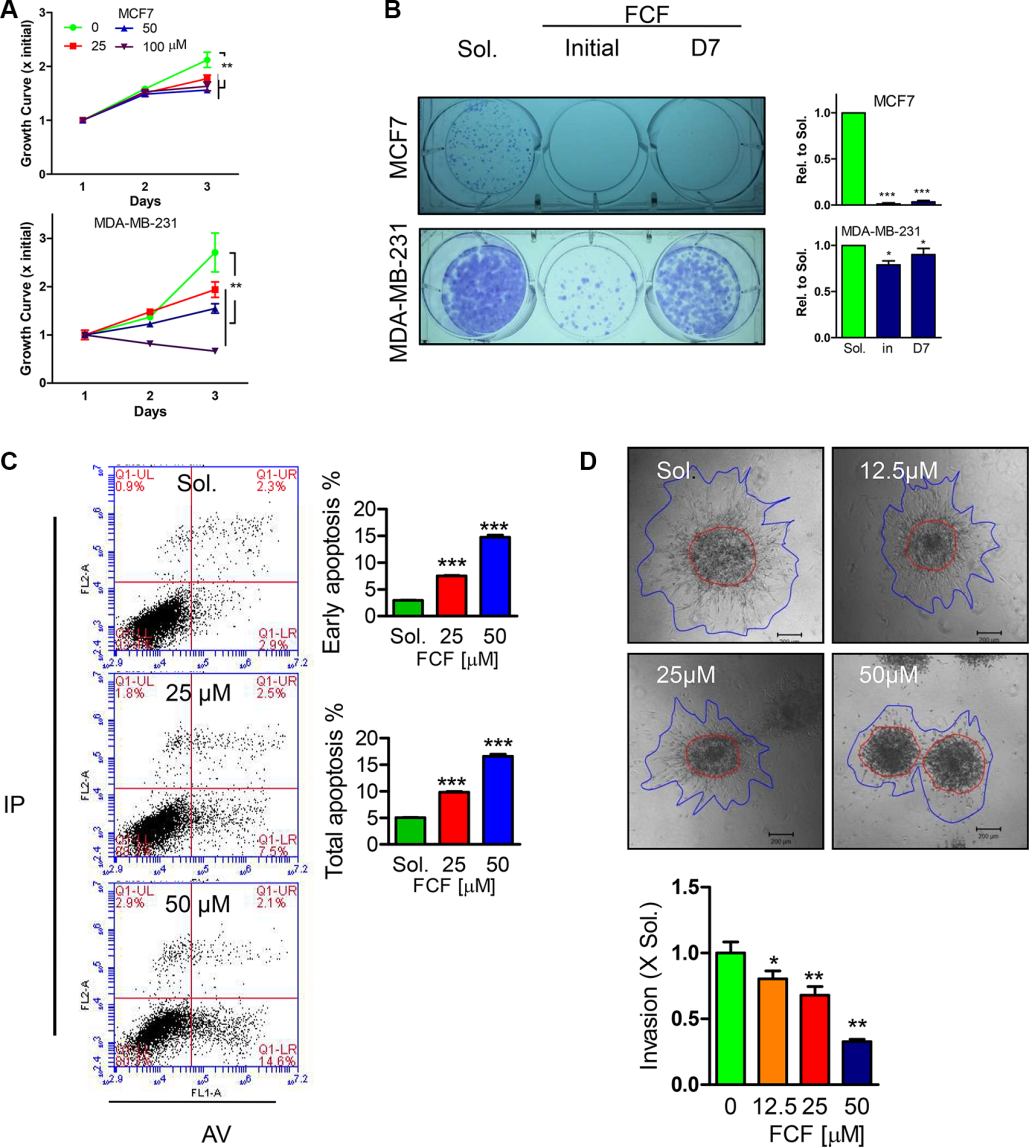
Effects of FCF on proliferation, single cell colony formation, apoptosis and invasion in BC cell lines (**A**) Different doses of FCF was used to inhibit septins in MCF7 and MDA-MB-231 cells with continued cultures for 3 days, the cell viability was detected by MTT assay. (**B**) The impact of FCF (50 μM) on single cell colony formation determined in MCF7 and MDA-MB-231 cells was evaluated after 14 days cultures, the initial and D7 indicate FCF treatment occurs right after or in 7 days after culture. (**C**) Apoptosis events were determined by FACS analysis after PI/AnnexinV staining in MDA-MB-231 cells treated with FCF for 48 hours. (**D**) The consequence of FCF on MDA-MB-231 cell invasion in collagen matrix for 24 hours. Data were obtained from 3–5 independent experiments, *p < 0.05, **p < 0.01, ***p < 0.001 versus solvent or control.

In order to generally evaluate the importance of septin proteins in affacting breast cancer (BC) cells proliferation and invasion, Forchlorfenuron (FCF), a specific conventional inhibitor for septin proteins [5, 12], was firstly applied to BC cells, MDA-MB-231 and MCF7. Within 3 days treatment, FCF displayed dose dependent inhibition on MDA-MB-231 and MCF7 proliferation determined by MTT assay (Figure 1A). Of note, 100 μM FCF was able to induce cytotoxicity as seen by the continually depressed cell viability and herein we excluded the dose in the following tests to avoid false positive results. Similarly, the ability of single BC colonies formation was also greatly impaired by FCF treatment (50 μM) whenever the treatment initiated on day1 or day7 during 14 days cultures (Figure 1B). The link of septin inhibition with cellular apoptosis was detected with Annexin V/PI staining based FACS analysis, demonstrating FCF would does dependently enlarge early and total apoptotic cell population in MDA-MB-231 cell (Figure 1C). In the other hand, we developed a three dimensional culture assay in collagen matrix to evaluate BC cell invasive ability. With the increased doses, FCF was gradually suppressing MDA-MB-231 cell invading into collagen matrix within 24 hours (Figure 1D). Due to high levels of Septin proteins expression was discovered in Paclitaxel resistant MDA-MB-231 cells [18], we tested the effects of septin inhibition with FCF on Paclitaxel reduced cell growth, showing FCF would significantly but mildly enhance Paclitaxel efficiency in non-paclitaxel resistant MDA-MB-231 (Supplementary Figure S1A), the similar observation was also determined in SEPT2 and SEPT7 silenced cells (Supplementary Figure S1B).

### SEPT2 and SEPT7 expression and localization in BC cells

**Figure 2:**
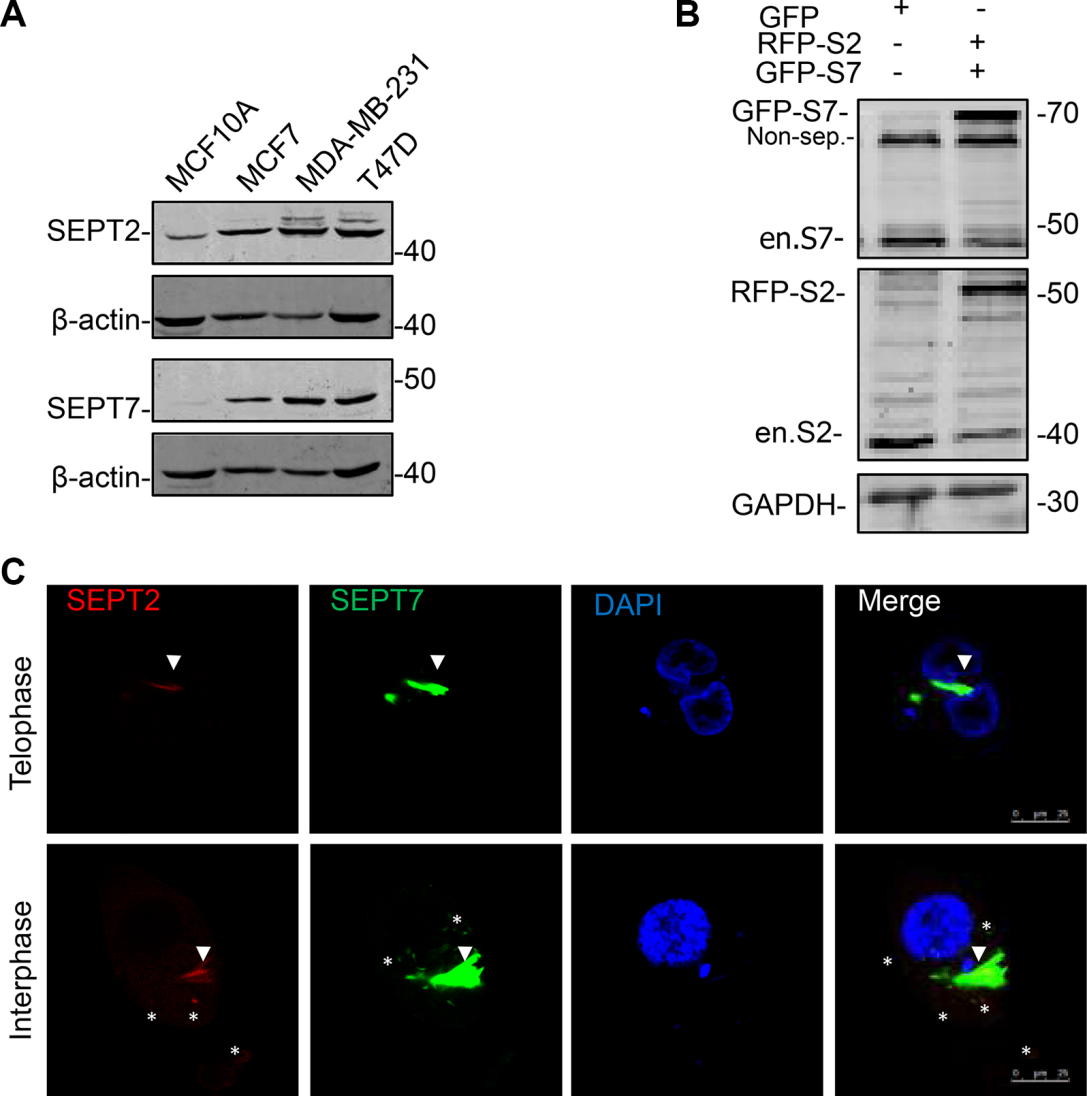
The expression and localization of SEPT2 and SEPT7 in BC cells (**A**) Western blotting showing protein expression levels of SEPT2 (S2) and SEPT7 (S7) in different types of breast cell lines, including MCF10A, MCF7, MDA-MB-231 and T47D. (**B**) After transient co-transfection of GFP-SEPT7 and mcherry (RFP)-SEPT2 constructs, the exogenous and endogenous expression of both proteins were determined in MDA-MB-231. Non-spc: non specific band. (**C**) Representative confocal microscope images showing the localization of SEPT2 and SEPT7 in MDA-MB-231 cells progressing in Interphase and Telophase. Red: RFP- SEPT2; Green: GFP- SEPT7; SEPT2: S2; SEPT7: S7; Blue: DAPI. Arrows and stars indicate the merged and non-merged regions separately.

Given FCF medicated septin dynamics inhibition greatly impaired BC biological behaviors; we thought core septin elements might be essential to explain how importance of septin proteins contribute to BC development. Herein, we decided to focus on SEPT2 and SEPT7 as they are basic elements required for structure organization and conceptually may contribute to septin biological functions. We detected their protein expression in different BC cells by western blotting (Figure 2A), revealing levels of both SEPT2 and SEPT7 were obviously higher in detected breast cancer cell lines in comparing with normal breast cell line MCF10A, especially in high invasive cell line, MDA-MB-231. To further visualize SEPT2 and SEPT7 subcellular distributions, we co-overexpressed SEPT2 (RFP tagged) and SEPT7 (GFP tagged) in MDA-MB-231 cells (Figure 2B) and observed a well co-localization of them was discovered in cleavage ring in telophase cells. However, although the co-localization pattern was also detectable in interphase cells, SEPT2 seemed not merely joining with SEPT7 to form perinucleus filaments; its scattered distribution, apart from SEPT7, was also discovered in the cytoplasma (Figure 2C).

### Reduced SEPT2 and SEPT7 expression inhibits BC proliferation, migration and invasion

**Figure 3:**
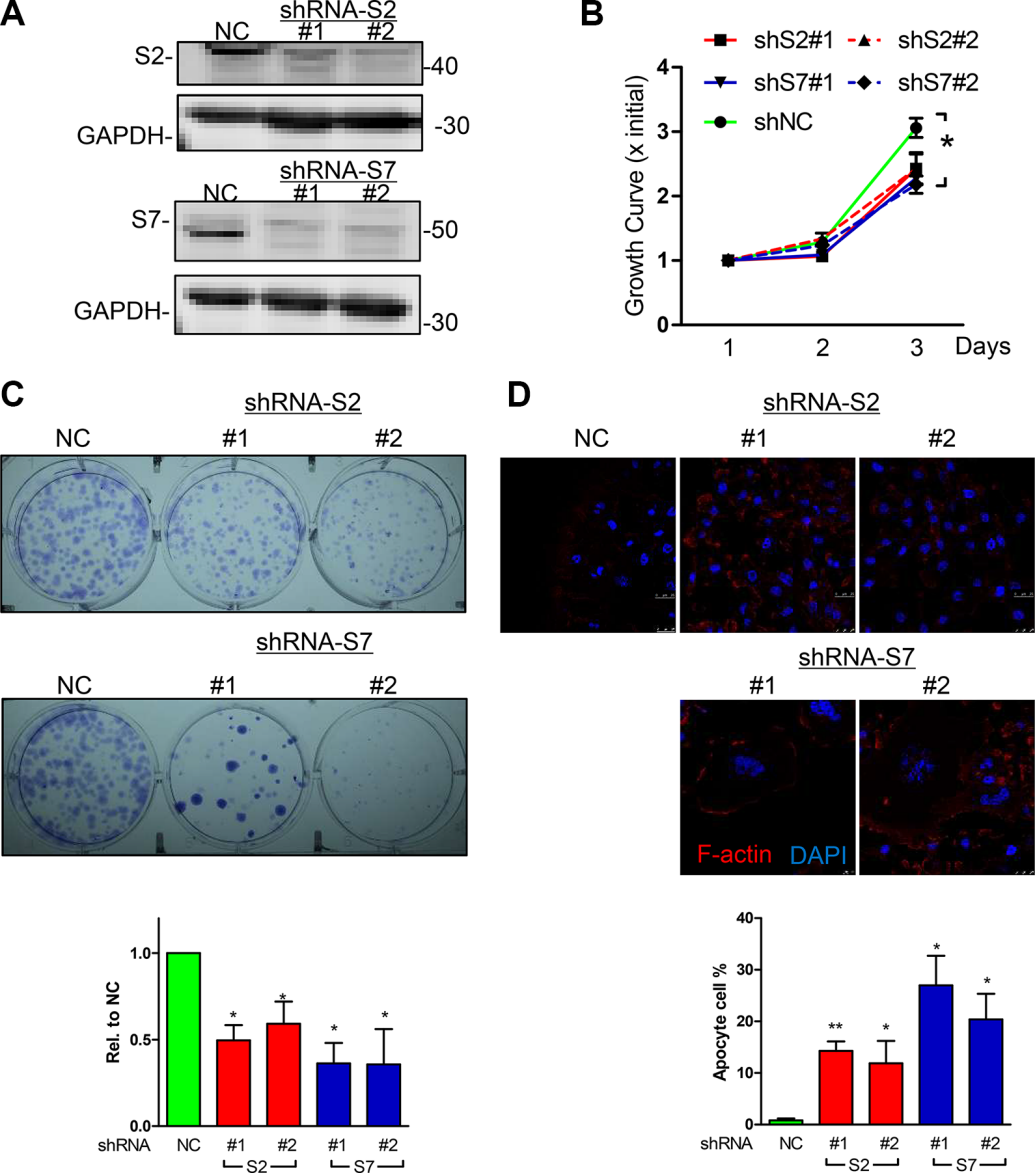
Effects of knockdown for SEPT2 and SEPT7 on BC cell proliferation (**A**) The efficacy of shRNA mediated stable knockdown against S2 and 7 (two shRNA colonies for each gene) was detected by western blotting in MDA-MB-231 cells. The corresponding consequences on (**B**) Cell proliferation was detected by MTT assay as well as (**C**) signal cell colony formation assay. (**D**) Cell morphology changes were visualized under confocal microscope (Blue, DAPI; Red, F-actin); Data were obtained from 3–5 independent experiments, *p < 0.05, **p < 0.01 versus shNC.

**Figure 4:**
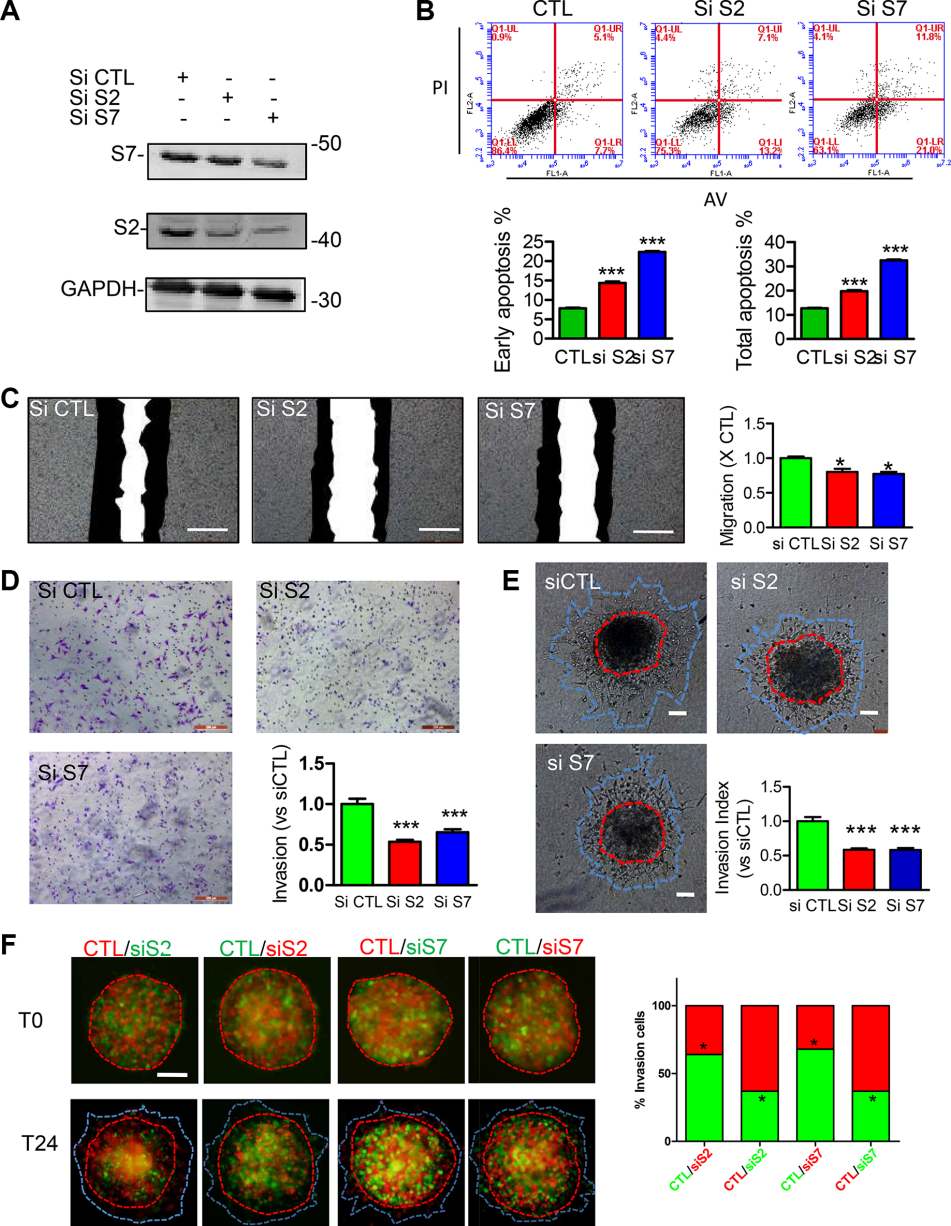
Effects of acute knockdown for SEPT2 and SEPT7 on BC cell apoptosis, migration and invasion (**A**) The efficacy of siRNA mediated transient knockdown of S2 and S7 in MDA-MB-231 cells was detected by western blotting. The corresponding influences on (**B**) cell apoptosis was assessed with PI/AnnexinV staining; (**C**) cell migration was evaluated in a scratch wound healing assay, scale bar = 50 μm; cell invasive ability was determined with (**D**) transwell based invasion though collagen as well as (**E**) 3D based tumor spheroids invasion after being embedded into a collagen matrix, scale bar = 200 μm; (**F**) A modified competitive invasion assay was evaluated by calculating ratios of forward invasive cells from tumor spheroid comprising equal amount of GFP or RFP labeled cells after control or siRNA treatment. Images were recorded under fluorescence microscope. Red line, the initial area; blue line, boarder of invasive cells, scale bar = 200 μm. Data were obtained from 4–5 independent experiments, *p < 0.05, **p < 0.01 versus control.

Loss of function for SEPT2 and SEPT7 expression were firstly conducted by generating stable knockdown cell line in MDA-MB-231 via pLKO-shRNA system (Figure 3A), which gave rise to a significant reduction in cell proliferation rate (Figure 3B) and single cell colonygenic ability (Figure 3C). Besides, SEPT7 silenced cells was able to induced a dramatic multi-nucleus accumulation as well as enlarged cell surface area, however, the phenotype was far less pronounced when SEPT2 was depleted (Figure 3D). In addition, the similar observation was also witnessed in SEPT2 or SEPT7 silenced MCF7 cells (Supplementary Figure S2). Meanwhile, acute knockdown the proteins expression of SEPT2 or SEPT7 (Figure 4A) significantly elevate early and total apoptotic rates in MDA-MB-231 cells (Figure 4B). In addition, reduced SEPT2 and SEPT7 expression were able to significantly decrease cell migration (Figure 4C) in a scratch wound healing assay. More consistently, SEPT2 and SEPT7 silenced cells performed less invasive abilities into collagen matrix (Figure 4D and 4E); we further confirmed the results by a competitive invasion assay via mixing equal amount of SEPT2 or SEPT7 silenced cell (Green or Red) with control siRNA treated cells (Red or Green), showing reduction in either SEPT2 or SEPT7 protein would induce remarkable suppression on BC cell invasive potentials(Figure 4F).

### Enforced expression of SEPT2 and SEPT7 promotes BC migration and invasion

**Figure 5:**
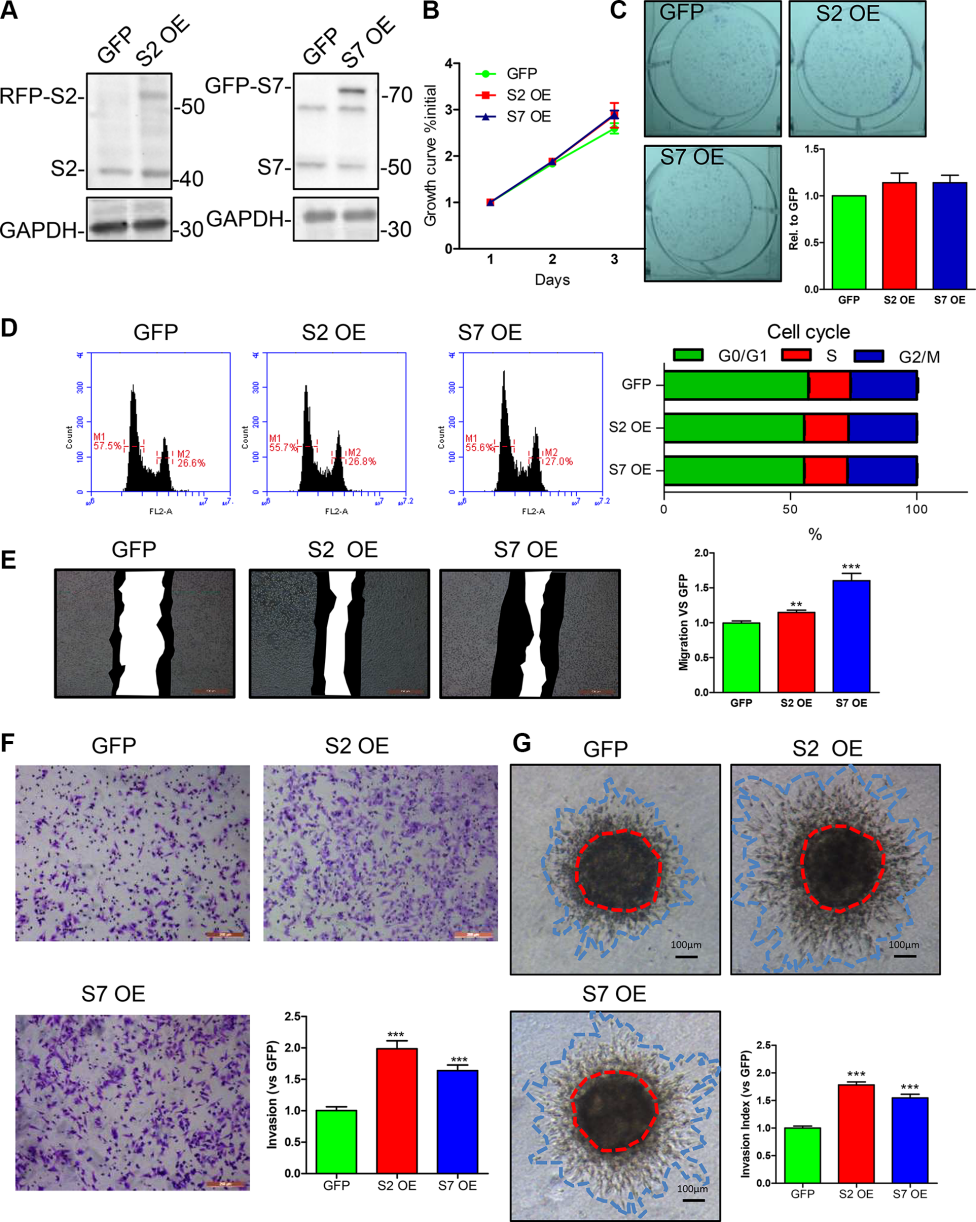
Effects of SEPT2 and SEPT7 overexpression on proliferation, cell cycle, migration and invasion in MDA-MB-231 cells (**A**) Stable transfected MDA-MB-231 cell lines with enforced expression of GFP, SEPT2 (S2 OE) and SEPT7 (S7 OE) were established andthe proteins expression were verified by western blotting. The corresponding influences on (**B**) proliferation was assessed by growth curves determined within 3 days; (**C**) the ability of single cell colony formation; (**D**) cell cycle progression detected by FACS analysis; (**E**) migration via scratch wound assay and the black region indicates the area migrated within 24 hours; the capability of cell invasion into collagen matrix was evaluated by (**F**) transwell based invasion assay and (**G**) 3D based tumor spheroids invasion. All data were obtained from 3–5 independent experiments, *p < 0.05, **p < 0.01, ***p < 0.001 versus GFP.

To further analyze the consequence of SEPT2 and SEPT7 overexpression on BC proliferation, migration as well as invasion, MDA-MB-231 cell lines with stable overexpressing RFP-SEPT2 or GFP-SEPT7 were established separately. In next, we found the enforced expression of the both septins (Figure 5A) had tiny increasing effects on BC cell proliferation (Figure 5B) and single colony formation (Figure 5C), but failed in reaching to significant difference. Besides, an identical result was obtained when determining cell cycle that demonstrated exogenously expressed SEPT2 and SEPT7 were not able to alter cycle progression (Figure 5D). However, cell motility was greatly elevated in SEPT2 and SEPT7 overexpressing cells assessed by a scratch wound healing assay (Figure 5E). Moreover, the ability of invasion into or through collagen matrix was also remarkably enhanced (Figure 5F and 5G).

### SEPT2 and SEPT7 are required for MEK/ERK activation

**Figure 6:**
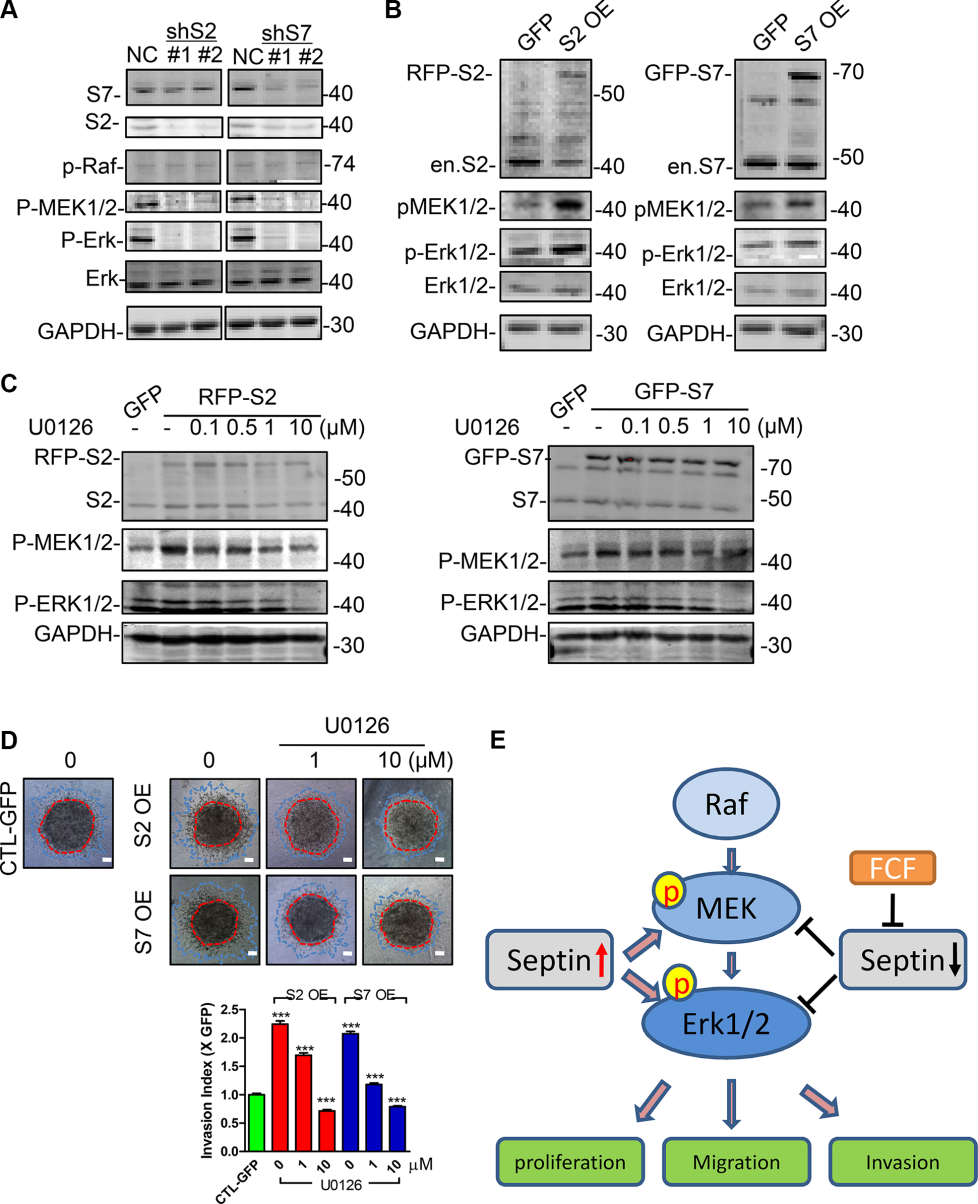
SEPT2 and SEPT7 were required for MEK/ERK activation in MDA-MB-231 cells The western blotting showing (**A**) the activation status of Raf/MEK/ERK in S2 or S7 depleted and (**B**) ectopic expressed MDA-MB-231 cells. (**C**) The effects of U0126 on MEK and ERK phosphorylation in S2 and S7 overexpressing cells and the consequent influences on (**D**) invasive capability. Red line, the initial area; blue line, boarder of invasive cells, scale bar = 200 μm. (**E**) The proposed working mechanism indicating the importance of Septins in regulating BC proliferation, migration and invasion. Pharmacological inhibition with FCF towards septin or SEPT2 and SEPT7 gene silencing would suppress MEK/ERK1/2 activation; high levels of Septins would elevate MEK/ERK1/2 activities, therefore promote breast cancer cell proliferation, migration and invasion. Data were obtained from 4–5 independent experiments, ***p < 0.001, versus solvent in control group.

Though the link of HIF1α protein stability and septin has been described previously [21–23], we found there was no clear influence on Deferoxamine (DFO) induced HIF1α protein expression in MDA-MB-231 cell treated with FCF or SEPT2 and SEPT7 silencing (Supplementary Figure S3A and S3B). Therefore, we switched our attention to other key pathways governing BC cell proliferation, migration and invasion such as MAPK, AKT signaling. Although there is no any influence on P38, JNK and AKT activation (Supplementary Figure S4A), SEPT2 or SEPT7 depletion would specifically impair ERK1/2 phosphorylation (Figure 6A), which was also detected in MCF7 cells (Supplementary Figure S4B). Therefore, we further examined the active status of its upstream kinases and found MEK1/2 but not Raf was inactivated in SEPT2 and SEPT7 depleted cells. Moreover, we further demonstrated inhibition of septin dynamics via FCF would also cause suppression towards ERK1/2 phosphorylation (Supplementary Figure S5) and but without any influence on SEPT2 and SEPT7 expression (Supplementary Figure S6A).

### Partial correction of enhanced invasion in SEPT2 and SEPT7 overexpressing cells via MEK inhibition

On the contrary to SEPT2 or SEPT7 silencing impaired MEK/ERK phosphorylation, we detected the phosphorylation was improved in SEPT2 or SEPT7 overexpressing cells (Figure 6B). To functionally verify the notion that SEPT2/7 was required for MEK/ERK activation, the both phosphorylation levels of MEK and ERK1/2 were corrected to level appeared in control cell by using a rang doses of U0126 (MEK inhibitor) in SEPT2 and SEPT7 overexpressing cells (Figure 6C), resulting in a partially suppressed invasive performance as the similar levels as observed in control cell (Figure 6D).

## DISCUSSION

In the current study, we revealed the essential role of septins for the proliferation and invasion of breast cancer cells. Generally, our results confirmed that the pharmaceutical inhibition of septin family displayed an apparent decrease in cell proliferation, single cell colony progression and invasion into collagen matrix. In particular, we selected the two core septin family members SEPT2 and SEPT7, and interrogated in detail their contribution to the malignant behavior of cancer cells by gain-and loss-of-function studies. Besides, our further data showed that these two septins served as oncogenes to participate in regulating breast cancer cell phenotypes through activating ERK signaling pathway.

Previous studies showed that different members of septin family have been implicated in carcinogenesis [1]. Due to the up to 11 mammalian septins expressed in breast cancer [10], it is necessary to evaluate whether the global inhibition of septin dynamics would affect malignant behavior of breast cancer cells. FCF was chosen and applied to inhibit septins, since it has been proven successfully for reflecting the regulatory functions of septins in other tumor cell lines, such as PC3, HCT116, and Hela [5, 12]. Of note, although some studies have demonstrated the efficacy of FCF as a useful tool for suppression of cell migration and proliferation [5], the effective doses in individual cell lines are variable. As such, we determined the optimal working doses (no more than 50 μM) in breast cancer cells, which could avoid the false positive experimental phenomenon from high dose induced cytotoxicity. On the basis of phenotypic changes detected by cell viability and invasiveness assays in combination with our newly developed experimental model mimicking tumor mass invasion procedure within collage matrix, we revealed that FCF could greatly suppress breast cell proliferation and invasion, which is consistent with the observation in Hela or MDCK cells [5]. These results indicated the dynamics of septin proteins is required for modulating malignant behavior of breast cancer cells.

A key characteristic of the mammalian septin family is its ability to form stable six-subunit core heteromer complexes, which are mainly orchestrated by SEPT2/6/7 [1, 3]. Furthermore, FCF was demonstrated to exert its effects on septin organization and dynamics by disrupting SEPT2/6/7 assembly [5]. Although SEPT6 is definitely crucial for Septin organization, its currently biological functions are far less clear and mainly associated with neuron development [24, 25], the other two core members would be more representative to be focused to explore the role of septin proteins in breast cancer biology as plenty of investigation discovered the importance of SEPT2 and SEPT7 within human cancer development [15, 26–30]. Putting all these important features together, resulted in our decision to select SEPT2 and SEPT7 as interesting molecules for further investigation of their specific contribution to malignant phenotypes of breast cancer cells. Indeed, the high expression levels of the both proteins was determined in breast cancer cell lines comparing with normal breast cell line MCF10A, revealing a potential positive links of SEPT2 and SEPT10 with breast cancer malignant properties. Considering that the protein's function is usually related to its subcellular localization, we first determined the distribution of the both septin proteins in breast cancer cells by double immunofluorescence analysis. Intriguingly, their localization seems quite disparate, supportive of a generally accepted notion that individual members of the septin family may display a rather variable subcellular expression depending on the cell type [9, 31]. Specifically, though co-staining of SEPT2 and SEPT7 was discovered in filaments structures surround nucleus, SEPT2 was localized to spot structures evenly scattered within the cytoplasm, which do not resemble the stress fibers as described in U373 and CHO-K1 cells [27, 31, 32], whereas SEPT7 was clearly detected in filament skeleton structures around the nucleus somewhat similar to the staining pattern in the U373 cell line [31]. Moreover, we found that both of SEPT2 and SEPT7 showed the differential localization in a cell cycle-dependent manner in highly invasive breast cancer cell line MDA-MB-231. These findings were reminiscent of those described in the human glioma cells [15, 31], suggesting that SEPT2 and SEPT7 may have evolved to fulfill multiple roles in dividing and non-dividing cells in addition to the best-known functions in cytokinesis [9].

Although the aberrant expression of SEPT2 and SEPT7 had been found to associate with various human cancer types including glioma, hepatoma, renal cell carcinoma, leukemia, and lymphoma [2, 10, 15, 30], there are only limited studies systematically evaluating their functional roles in tumoigenesis. SEPT2 was confirmed to be upregulated in hepatoma carcinoma cells (HCC) and its phosphorylation occurred on Ser218 is crucial to the HCC proliferation [29]. By contrast, SEPT7 was downregulated in human gliomas and overexpression of SEPT7 could suppress glioma cell growth [15]. In this study, we demonstrated that SEPT2 and SEPT7 had virtually identical promoting effects on breast cancer cell proliferation, migration and invasion. For their contribution to proliferation, we observed that SEPT2/7 depletion significantly decreased the cell proliferation rate and single cell renewal, which were, however, not affected in SEPT2/7-overexpressing breast cancer cells. This might be explained by endogenously expressed protein levels sufficient for the maintenance of the breast cancer cell proliferation. When investigating on migration and invasion by gain- and loss-of-function approaches, we can draw the general conclusion that both of SEPT2 and SEPT7 act as positive regulators of the breast cancer cell malignant behavior related to cell motility. This supports our expectations, because the septins have been shown to physically interact and cooperate with other cytoskeletal proteins such as actin, tubulin, and myosin II [27, 33], which directly or indirectly take part in the cell motility.

In this study, we found an interesting phenomenon that silenced SEPT7 protein expression was able to reduced SEPT2 protein levels. We have excluded the mismatching or off-target possibilities from siRNA or shRNA approaches after complementary sequence comparison between cDNAs of SEPT2 and SEPT7. Actually the dominate role of SEPT 7 protein in controlling other septin family member expression have been discovered in Hela [34] with shRNA approach and Sept7 genetic depleted mouse fibroblast cells [35], our data further confirm those previous discoveries in breast cancer cell lines and additionally in primary human umbilical vein endothelial cell (Supplementary Figure 6B). However, how SEPT7 influence other septins has not been experimentally explained by far. Considering the ubiquitous expression pattern of SEPT7 among tissue and as core septin element, we deduce it might be a fundamental molecule which protect or stabilize other Septin proteins during high-order skeleton structure constitution. The other possibility might link to the resistance of the septin complex to protein degradation. Once the depletion of SEPT7, the key septin, the high ordered septin architecture would be insufficiently constituted and ubiquitously degradation might occur.

Uncovering the molecular mechanisms underlying the cell biological functions of SEPT2 and SEPT7 is a crucial issue in our study. Given that some of key signaling pathways, such as HIF1α, PI3K/AKT and MAPK/ERK1/2 pathways make a substantial contribution to govern proliferative, migrating and invasive phenotypes in breast cancer cells [36–39], we considered whether there was any correlation between SEPT2/7 functions and these pathways. Our results confirmed that SEPT2 or SEPT7 depletion would reduce levels of phospho-ERK1/2 and phospho-MEK1/2; however, levels of phospho-AKT, phospho-Raf, phosoho-JNK and phospho-p38 remained unchanged. This suggests both SEPT2 and SEPT7 regulated breast cancer cell biological behavior through the activation of the ERK pathway to some extent. It is interesting to note that pharmaceutical inhibition of septin dynamics would suppress ERK activities as well, implying the possible involvement of additional septins in synergistically boosting ERK signaling. Indeed, many studies have demonstrated that SEPT9 functions as an oncogene in breast cancer cell lines and its overexpression leads to accelerated growth kinetics, increased cell motility and promoted invasion [1, 22]. Moreover, SEPT9 can bind to SEPT2 and SEPT7 in anon-stoichiometric manner, whereby long isoforms of SEPT9 stabilize the formation of higher-order oligomers [40, 41]. In this regard, future study is needed to examine whether SEPT9 plays a role in activating ERK pathway. Enforced expression of SEPT2 and SEPT7 would significantly increase levels of phospho-ERK1/2 and phospho-MEK1/2. On this basis, pharmaceutical inhibition of MEK via U0126 not only normalized MEK and ERK phosphorylation but also reversed invasive potential of SEPT2/7-overexpressing cells. Due to a similar biological behaviors as well as cellular interaction of SEPT2 and SEPT7 in breast cancer cells, we suggested the septin core elements; at least SEPT2 and SEPT7, are necessary for septin biological performance. Our data demonstrated once deficient in any of both would impairs BC malignant behavior and we deduced it is possible that an even greater BC suppressive effects might be achieved after reduction of both genes. How Septins maintain MEK/ERK activation is an interesting issue. Given inhibition of Septin dynamics was also able to disturb activation of MEK/ERK, we speculate Septin formed skeleton scaffold structures and dynamics would be sufficient and facilitate MEK/ERK phosphorylation. Taken together, our data revealed a novel regulation mechanism that at least two septins SEPT2 and SEPT7 were required for MEK/ERK activation in breast cancer cells.

In summary, our study discloses both SEPT2 and SEPT7 are essential for breast cancer cell migration and invasion by controlling MEK/ERK MAPKs activation and also suggests the targeting septin proteins might be a novel direction for breast cancer therapy.

## MATERIALS AND METHODS

### Antibodies

Antibodies against SPET2 (#11397-1-AP) was from proteintech, SPET7 (#ABT354) was from Millipore; Akt (#46910), p-Akt (Ser 473, #4060P), p-MEK1/2 (#2338), p-Erk1/2 (#4370P), ERK (#137F5), p-p38 MAPK (Thr180/Tyr182, #4511P), SAPK/JNK (#9252P), p-SAPK/JNK (#9242P) were from Cell signaling technology. Forchlorfenuron (#32974) and Deferoxamine (#D9533) was purchased from Sigma; U0126 (#9910) was from Cell signaling technology. Secondary antibodies of goat anti-rabbit IgG (#A23920) and goat anti-mouse (#A23710) were from Abbkine. Rat tail collagen type I (#354236) was from Corning.

### RT-PCR and plasmid construction

Total RNAs were isolated by miRNA kit (#DP501, Tiangen) and cDNAs were generated by reverse transcription kit (#KR-106-02, Tiangen). *SEPT2* and *SEPT7* coding region were amplified using the cDNAs as template with Tag polymerase (#11304-029, Invitrogen) and cloned into pmcherry-C2 and pEGFP-C1 in a frame with RFP and EGFP respectively, and were verified by sequencing. DNA sequences of RFP-SEPT2 and EGFP-SEPT7 were subcloned into pCDH-CMV-puro plasmid by using Nhe I/ BamH I. The primer sequence and restriction enzyme were listed in Table 1.

**Table 1:** Sequences of DNA primers and siRNAs

	PCR primer sequence	Restriction enzyme site
*SEPT7* forward	aagcttcgatgtcggtcagtgcgagatccgctgctgctgaggagaggagcgt	Hind III
*SEPT7* reverse	ggtaccttaaaagatcttccctt	Kpn I
*SEPT2* forward	agcgctcgagcgccaccatgtctcgattctacgatgtctaagc	Xho I
*SEPT2* reverse	agcgagaattcttacacgtggtgcccgagag	EcoR I
	siRNA forward sequence (5′–3′)	siRNA reverse sequence (5′–3′)
siRNA against *SEPT2*	gcugcucacaaucguugautt	auaacuauugugagcagctt
siRNA against *SEPT7*	gcugcucacaauaguugatt	aucaacuauugugagcagctt

### Cell cultures

MDA-MB-231, MCF7, T47D and HEK293T were purchased from the American Type Culture Collection (ATCC, Manassas, VA, USA). HEK293T, T-47D and MDA-MB-231 cells were cultured in Dulbecco's Modified Eagle Medium (DMEM, GIBCO) supplemented with 10% fetal bovine serum (FCS, GIBCO); MCF7 cells were cultured in minimal essential medium (MEM, GIBCO) Primary human umbilical cord endothelial cells (HUVEC) were isolated as [42] described. HUVEC and MCF10A cells were cultured in M199 medium (GIBCO) with 20% FBS. All cells were cultured in medium supplemented penicillin (50 U/mL) and streptomycin (50 μg/mL) and maintained in incubator with 5% CO_2_ at 37°C.

### Lenti-virus generation and infection

The pLKO.1 shRNA lenti-virus system was used to generate shRNA virus against Human *SEPT2* and *SEPT7*. The shRNA plasmids against human *SEPT2* (#1: TRCN0000062153;#2: TRCN0000062154) and *SEPT7* (#1 TRCN0000146634; #2 TRCN0000146635) were purchased from Sigma. The pCDH-CMV-EF1-puro lenti-virus system was utilized to generate human RFP-SEPT2 and GFP-SEPT7 virus. The packing procedures were as previously described [43]. Virus infection was conducted by incubated virus solution with cells for 48 hours and positive cell population was obtained by puromycine (3 μg/mL) selection for 7 days.

### siRNA transfection

Lipofectamine2000 (#52758, Invitrogen) was used for the siRNA transfection according to the manufacture instructions. The siRNA sequences were synthesized by Genepharma and liseted in Table 1.

### Proliferation assay

Cell viability was measured using the 3-(4,5-dimethylthiazol-2-yl)-2,5-diphenyltetrazolium bromide (MTT, #M2003, Sigma) cell-metabolism assay. Cells were seeded in 96-well plates at 5000 cells in 200 μL culture medium, followed by FCF treatment with indicated concentration for up to three days. 0.1% ethanol was used as a control. Twenty μL of MTT were added to each well and incubated for 4 h at 37°C. After removing medium, 200 μLof dimethylsulfoxide (DMSO) were added to each well. Cell viability was assessed using the microplate reader (Enspire2300, Perkin Elmer) at 595 nm absorbance.

### Single cell colony formation assay

Cell suspension at a density (1000 cells/mL) was transferred into 6-well plates (2000 cells per well) and cultured for 14 day, followed by staining with 0.1% crystal violet (#V5265, Sigma) solution for 10 min. Cell colonies were recorded by digital camera after intensive washing with water at room temperature. The images were evaluated by the colony coverage analyzed with Image J software.

### Immunoflorescence

Cells were fixed with 4% polyformalin for 10 min at RT, and blocked with 0.1% BAS in PBST for 10 min and then incubated with pholloidin-TRITC (1:1000 dilution, #R450, Thermo Fisher) overnight at 4°C. Finally, the nucleus was stained with 5 μg/mL DAPI for 5 min. The cells were washed three times with ice-cold PBS for 5 min each between every two procedures above. The images were taken by fluorescence microscope (DMI 4000B, Leica) or confocal microscopy (TCSSP5II, Leica).

### Scratch wound assay

Monolayer cells were wounded with a sterile pipette tip to generate a crossing cell-free space. After three times rising with PBS, cells were allowed to grow in culture medium for 24 hours. The images at initial and ending time points were recorded with microscopy (DMI4000B, Leica), and the migrated area was calculated as cell free space (SFS) at initial time minus SFS at ending time. The SFS was measured by using Image J Software.

### Apoptosis and cell cycle determination

Cell suspensions in PBS were freshly prepared and stained with PI/Annexin V kit (#KGA108, KeyGEN BioTECH) for apoptosis determination or stained with DAPI for cell cycle assessment and followed by analyzing with Accuric6 (BD).

### Three-dimensional (3D) spheroid invasion assay

The assay was modified from our previously report [42]. Briefly, cells spheroids were generated by hung-drop cultures of 2000 cell in 25 μL culture medium containing 20% methylcellulose (v/v) in surface non-treated plates (#TCD00035, BIOFIL) for 24 hours. Spheroids were collected with PBS containing 10% FBS and transfer to in a 15 mL tube, following by spheroids sink down by gravity for 10 min. After removal of medium, spheroids were embedded in collagen matrix (1.5 mg/mL, pH7.4 buffered in 1 × M199) in RT for 30 min until matrix polymerization. Culture mediums containing solvent or compound were filled and spheroids were further cultured for another 24 hours. Spheroids images were recorded in the indicated time points and area was measured with Image J software. The invasive performance was assessed by Invasion Index calculated as the ratio of (Area end - Area initial)/Area initial.

### Western blot

Western blot was performed as our previously publication [42]. Briefly, Cells were harvested after twice rising with PBS and then lysed with 1× SDS loading buffer. Samples were electrophoresed on 10% SDS-polyacrylamide gels, and transferred to NC membranes. The membranes were blocked for 1 h with 3% (w/v) BSA, incubated with the primary antibodies overnight at 4°C and the secondary antibodies for 1 h at room temperature. Antigen–antibody complexes were detected by Odyssey CLx image system (LI-COR).

### Statistics

All data are generated from at least three times independent assays and presented as mean ± S.E.M. Statistical analysis of the data for multiple comparisons were performed by analysis of variance (ANOVA). For single comparison, the significance of differences was determined by *t*-test. *P < 0.05, **P < 0.01; ***P < 0.001.

## SUPPLEMENTARY MATERIALS FIGURES


