# Supplementary material for: The requirement of SEPT2 and SEPT7 for migration and invasion in human breast cancer via MEK/ERK activation
Source: Oncotarget. 2016 Aug 19;7(38):61587–600. doi: 10.18632/oncotarget.11402 (PMC5308674; doi:10.18632/oncotarget.11402)
Supplement: Supplementary file 1 [file oncotarget-07-61587-s001.pdf]

The requirement of SEPT2 and SEPT7 for migration and invasion in human breast cancer via MEK/ERK activation

Supplementary Materials

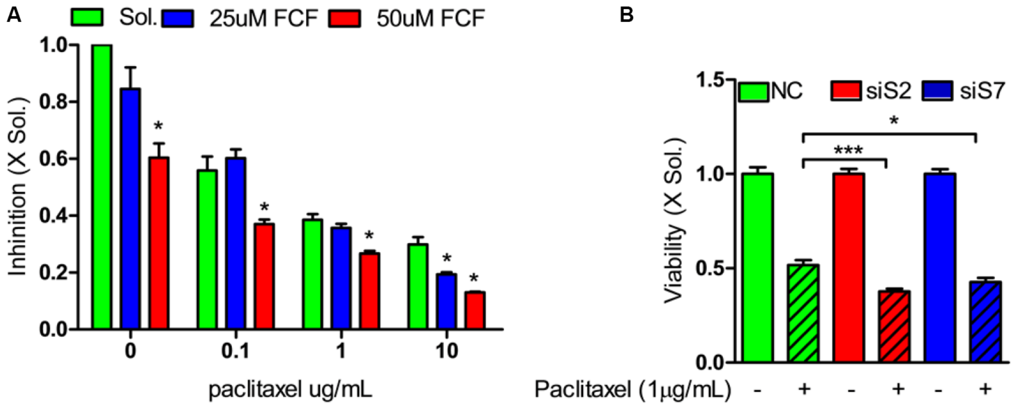

Supplementary Figure S1: The effects of FCF or SEPT2 and SEPT7 silence on paclitaxel inhibited cell growth. (**A**) MDA-MB-231 cells were incubated with different does of paclitaxel combined with FCF for 48 hours and cell cell viability was measured by MTT assay. (**B**) The responsed of MDA-MB-231 cells with siRNA mediated SEPT2 and SEPT7 silence to paclitaxel (1 μg/mL) caused cell growth inhibition were assessed by MTT assay.

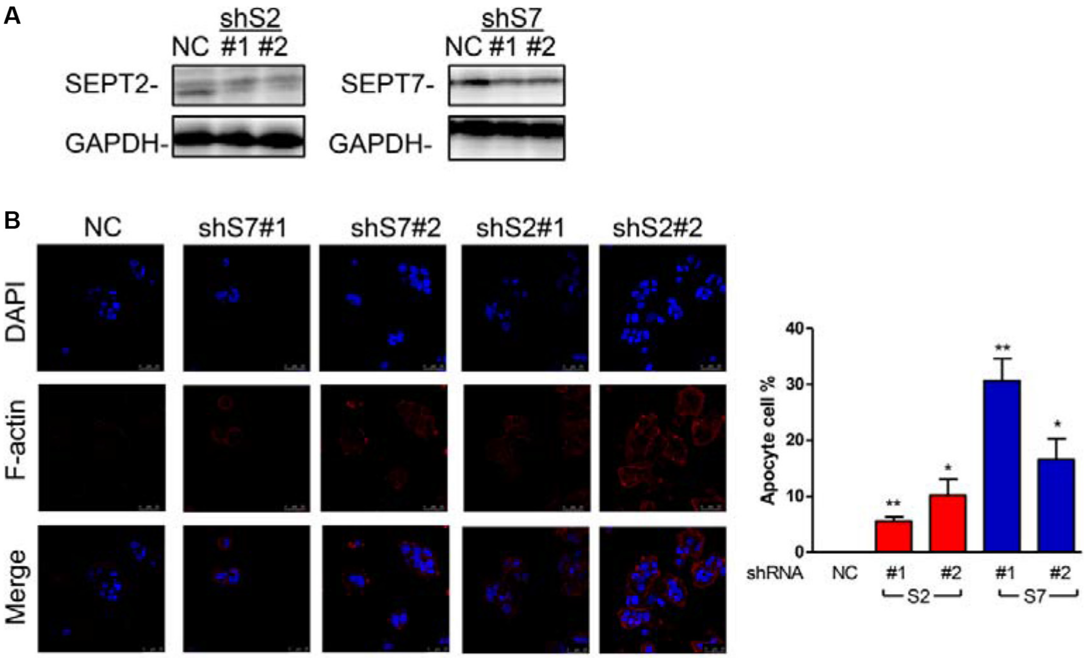

Supplementary Figure S2: The effects of SEPT2 and SEPT7 silence on MCF7 cell apocyte phenomenon. (**A**) Western blotting showing the shRNA efficiency suppressed S2 and S7 protein expression in MCF7 cells and (**B**) the consequent multi-nucleus events were visualized under confocal microscope (Blue, DAPI; Red, F-actin). The apocyte rate was summarized from at least 4 times independent experiments, *$P < 0.05$, **$P < 0.01$ versus NC.

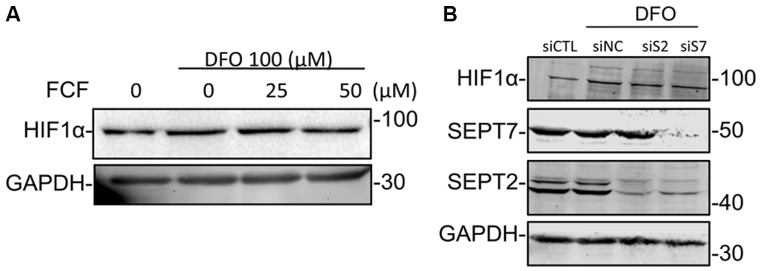

Supplementary Figure S3: The consequence of FCF or SEPT2 and SEPT7 silence on DFO induced HIF1α expression in MDA-MB-231 cells. Chemical hypoxia was conducted with treatment with 100 μM DFO and HIF1α expression were detected in (**A**) FCF treated or (**B**) SEPT2 and SEPT7 silenced MDA-MB-231 cells.

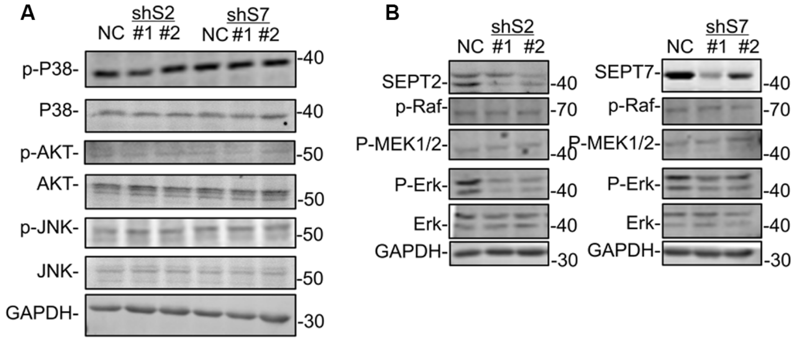

Supplementary Figure S4: Western blotting showing the effect of SEPT2 and SEPT7 knockdown on (A) phosphorylation levels of P38, JNK and AKT in MDA-MB-231 cell and (B) Raf, MEK, ERK phosphorylation in MCF7 cells.

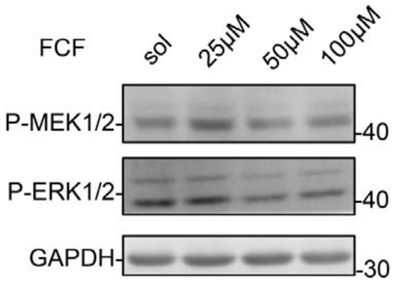

Supplementary Figure S5: Western blotting showing the changes of phosphorylation levels of MEK1/2 and ERK1/2 in MDA-MB-231 cells treated with indicated doses of FCF for 24 hours.

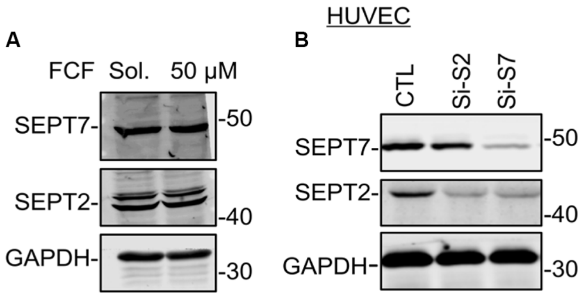

Supplementary Figure S6: The western blotting representing (A) the influence of FCF on SEPT2 and SEPT7 expression in MDA-MB-231 cells and (B) the altered proteins levels of SEPT2 and SEPT7 in primary human umbilical vein endothelial cells (HUVEC) after siRNA medicated silencing for each gene.
